# Development of the 12-Item Social Media Disinformation Scale and its Association With Social Media Addiction and Mental Health Related to COVID-19 in Tunisia: Survey-Based Pilot Case Study

**DOI:** 10.2196/27280

**Published:** 2021-06-09

**Authors:** Noomen Guelmami, Maher Ben Khalifa, Nasr Chalghaf, Jude Dzevela Kong, Tannoubi Amayra, Jianhong Wu, Fairouz Azaiez, Nicola Luigi Bragazzi

**Affiliations:** 1 Higher Institute of Sport and Physical Education of Kef, University of Jendouba Jendouba Tunisia; 2 Group for the Study of Development and Social Environment (GEDES) Faculty of Human and Social Science of Sfax Sfax Tunisia; 3 Postgraduate School of Public Health Department of Health Sciences (DISSAL) University of Genoa Genoa Italy; 4 Research and Applications Unit in Marketing (URAM), Faculty of Economics and Management of Tunis (FSEGT), University of Tunis El Manar (UTM) Tunis Tunisia; 5 Higher Institute of Sport and Physical Education of Sfax, University of Sfax Sfax Tunisia; 6 Department of Mathematics and Statistics York University Toronto, ON Canada

**Keywords:** COVID-19 pandemic, media disinformation, social media addiction, mental health, scale validation

## Abstract

**Background:**

In recent years, online disinformation has increased. Fake news has been spreading about the COVID-19 pandemic. Since January 2020, the culprits and antidotes to disinformation have been digital media and social media.

**Objective:**

Our study aimed to develop and test the psychometric properties of the 12-item Social Media Disinformation Scale (SMDS-12), which assesses the consumption, confidence, and sharing of information related to COVID-19 by social media users.

**Methods:**

A total of 874 subjects were recruited over two phases: the exploratory phase group had a mean age of 28.39 years (SD 9.32) and the confirmatory phase group had a mean age of 32.84 years (SD 12.72). Participants completed the SMDS-12, the Internet Addiction Test, the COVID-19 Fear Scale, and the 10-item Perceived Stress Scale. The SMDS-12 was initially tested by exploratory factor analysis and was subsequently tested by confirmatory factor analysis.

**Results:**

The test supported the three-factor structure. In addition, no items were removed from the measurement scale, with three factors explaining up to 73.72% of the total variance, and the items had a lambda factor loading ranging from 0.73 to 0.85. Subsequently, confirmatory factor analysis confirmed the robustness of the measure by referring to a wide range of goodness-of-fit indices that met the recommended standards. The construct validity of the scale was supported by its convergent and discriminant validity. The reliability of the instrument examined by means of three internal consistency indices, and the corrected item-total correlation, demonstrated that the three dimensions of the instrument were reliable: Cronbach α values were .89, .88, and .88 for the consumption, confidence, and sharing subscales, respectively. The corrected item-total correlation ranged from 0.70 to 0.78. The correlation of the instrument’s dimensions with internet addiction and mental health factors showed positive associations.

**Conclusions:**

The SMDS-12 can be reliably utilized to measure the credibility of social media disinformation and can be adapted to measure the credibility of disinformation in other contexts.

## Introduction

During the COVID-19 pandemic, caused by the emerging SARS-CoV-2, people around the world have been leaning toward an excessive use of the internet [1] and social media. This is the case because, on the one hand, this activity can lower their feelings of loneliness and, on the other hand, it can provide them with information on the states of emergency in their countries and globally [2].

This pandemic is characterized by a high potential for contagion, a low availability of vaccines, an absence of specifically effective drugs, and an exponential spread, which has impacted people’s lifestyles and led to feelings of insecurity [3,4], fear [5], and even community panics in several populations [6-10].

Almost everyone is interested in hearing reliable, updated information concerning the pandemic, vaccines, and anything related to COVID-19. This is because during the pandemic, in addition to seeing their usual activities restricted, people are exposed to a wide range of information, including official messages, as well as erroneous and misleading news from a range of unreliable sources [11,12]. The global spread of the COVID-19 pandemic has been reflected in the dissemination of misinformation on social media and conspiracy theories about its origins [13].

Indeed, since the beginning of the spread of the disease, several fake news items related to the outbreak have been shared on social networks. Examples include that the virus was caused by 5G cell phones, was deliberately disseminated for political or financial reasons, was a biological weapon, or was not more dangerous than influenza, with the threats being exaggerated as a way of limiting freedom [14].

Sharing false news that contains biased, emotionally charged information tends to capture more attention and interest than detached, positive, or neutral information [15]. Communication is of crucial importance in the control of outbreaks, and misinformation represents a major public health concern in that the use of social media as a means of keeping abreast of all the pandemic-related news is becoming very popular for several categories of people, due to its capacity of providing information in real time [16]. Likewise, social media can be utilized as platforms and venues for disseminating false information in times of crisis [16].

From another perspective, according to Alheneidi et al [17], besides information and communications technologies, psychosocial factors seem to play a key role. Personal negative feelings, such as loneliness, experienced during the COVID-19–induced lockdown have been shown to promote internet addiction behaviors, resulting in a significantly increased number of hours spent online. The study was conducted in two Arabic countries—Kuwait and the Kingdom of Saudi Arabia—and showed that people who experienced greater loneliness were more likely to consume pandemic-related news from social media.

Governments have been implementing behavioral strategies and nonpharmaceutical interventions (NPIs), including social and physical distancing and stay-at-home orders, to control the spread of COVID-19 and flatten the epidemic curve [18,19]. As a consequence, addiction to social media has increased, as it is the most accessible and easy-to-use means of communication and social interaction, resulting in excessive news consumption, which can lead to acute psychological distress and mental health problems, such as anxiety and depression [20].

The public health measures thus taken and enforced by governments, such as the compulsory wearing of masks, quarantine, mobility restrictions, social and physical distancing, the closures of several public places, bans on gatherings, partial curfews, and isolation of sick people, risk being compromised because of erroneous information constantly propagated on social media platforms. Indeed, Wang et al [21] have found that health-related misinformation is a very common phenomenon on social media and tends to be more prevalent than the diffusion of accurate information, in general.

As a matter of fact, significant amounts of disinformation and conspiracy theories have been disseminated through several social media platforms and consumed by users willing to learn about the COVID-19 pandemic. In general, the COVID-19 outbreak was accompanied by a large proliferation of fictitious and inaccurate information on the virus, which was spread, in particular, by social networks [22].

In a descriptive study by Cinelli et al [23] on the dissemination of COVID-19–related information on five social media platforms—Twitter, Instagram, YouTube, Reddit, and Gab—analyses highlighted a great amount of information about the COVID-19 outbreak disseminated on social networks, a large part of which was false information or disinformation.

COVID-19–related misinformation can bring not only high stress rates and serious mental consequences [24], but can also have a negative impact on the effectiveness of government strategies, such as the compulsory wearing of masks, confinement, and social and physical distancing. For instance, the false belief that the virus threat is being exaggerated may result in poor compliance and adherence to NPIs and, therefore, jeopardize the fight against the coronavirus. In the health field, dissemination of spurious news poses serious challenges because it can delay or prevent the delivery of effective care provisions or even threaten people’s lives.

Unfortunately, many fake news items are accepted by the general population. For instance, a recent US study on COVID-19 conspiracy speculation found that over 80% of participants surveyed believed a particular conspiracy theory to be “probably” or “certainly” true [25]. In another study conducted in the United States, Uscinski et al [26] found that 29% of subjects believed that the communication on COVID-19 was biased for political reasons, in order to place then–US President Donald Trump at a disadvantage against his competitors.

If false news is accepted as true, dissemination of scientifically proven and evidence-based narratives to amend such fake news would not have a significant impact on belief in disinformation [27].

Although governments, public health decision makers and policy makers, and other stakeholders are suffering from the dissemination and sharing of misinformation on social media, there exists no scale that enables the quantitative assessment of the behavior of social media users in the face of misinformation related to COVID-19.

Therefore, the objective of this study was to develop and validate an ad hoc measurement tool to measure the behavior of social media users in terms of consumption, credibility, and sharing of information related to COVID-19.

## Methods

### Ethical Declaration

The protocol for this study received approval from the Ethics Committee of the Higher Institute of Sport and Physical Education of Kef, University of Jendouba, Jendouba, Tunisia. The study protocol also received ethical authorization from the UNESCO (United Nations Educational, Scientific and Cultural Organization) Chair in Health Anthropology Biosphere and Healing Systems, University of Genoa, Genoa, Italy, as well as from the Higher Institute of Sport and Physical Education of Sfax, Sfax, Tunisia. The proposal was also approved by the Ethics Committee of the University of Jendouba. This study was undertaken in accordance with the ethical standards of the Declaration of Helsinki in 1964 and its subsequent amendments.

### Participants and Data Collection

A total of 874 subjects, with a mean age of 30.62 years (SD 11.37), who were recruited from social media platforms over two time periods participated in this study. Participants were interviewed by means of an online questionnaire distributed via two social media platforms: Facebook and Twitter. The characteristics of the participants (ie, gender, student or employment status, academic level, and marital status) are presented in Table 1.

**Table 1:** Sociodemographic characteristics of the participants selected for this study.

Characteristic		Value (N=874), n (%)
**Gender**		
	Male	415 (47.5)
	Female	459 (52.5)
**Student or employment status**		
	Student	297 (34.0)
	Public function employee	211 (24.1)
	Unemployed	94 (10.8)
	Private function employee	233 (26.7)
	Retired	39 (4.5)
**Academic level**		
	Secondary	252 (28.8)
	University	622 (71.2)
**Marital status**		
	Single	446 (51.0)
	Married	304 (34.8)
	Other	124 (14.2)

Study participants were randomly divided into two groups with the same number of individuals in each: one group participated in the exploratory phase and the other participated in the confirmatory phase.

The exploratory phase group consisted of 437 out of 874 (50.0%) participants, of which 248 (56.8%) were female and 189 (43.2%) were male; the mean age of the participants in this group was 28.39 years (SD 9.32). The confirmatory phase group consisted of 437 out of 874 (50.0%) participants, of which 211 (48.3%) were female and 226 (51.7%) were male; the mean age of the participants in this group was 32.84 years (SD 12.72).

### Instruments

#### Sociodemographic Questionnaire

The sociodemographic questionnaire consisted of questions about age, gender, level of education, the city in which the participant was currently living during the COVID-19–induced restrictions, student or employment status, and marital status.

#### Development of the 12-Item Social Media Disinformation Scale

A thorough review of the literature showed that information consumption includes a series of behaviors and processes, such as information seeking and information encounter (ie, *finding without seeking*). The first is defined as the intentional acquisition of information, while information encounter describes how individuals come across information without deliberately seeking or retrieving news [28].

Understanding social media consumption has proven to be a very important dimension to incorporate into the measurement instrument, as it can help analyze how people may face disinformation. The literature has shown that individuals who consume disinformation make a judgment on the credibility of the message, depending on the source of the information, the story, and the context [29]. Indeed, the work of Rosnow [30] has shown that if disinformation circulates repeatedly, it will be absorbed, reinforced, and accepted as credible.

A further step in the process of information consumption is news sharing. Previous studies have reported various personal predictors of sharing misinformation, such as fear of missing out, social media fatigue, lack of skills in verifying the reliability of information, and information overload on social media. When news about a rumor is collectively shared by communities, the dissemination of that message is amplified.

Based on these theoretical findings, we operationalized the measurement of disinformation through the 12-item Social Media Disinformation Scale (SMDS-12) instrument. The first dimension of the SMDS-12 assesses the degree to which COVID-19 information is consumed from social media. The second dimension reports users’ judgments about their degrees of belief, confidence, and trust in information related to COVID-19 shared on social media. The third dimension describes how one interacts with such news; in this case, sharing of information related to COVID-19.

Each dimension is made up of four items that are rated on a 5-point Likert scale, ranging from 1 (strongly disagree) to 5 (strongly agree).

Subsequently, a construct evaluation was carried out by a focus group made up of seven experts: two professionals in social networks, both administrator and content creators; two professors in human sciences; two experts in linguistics; and an expert in information and communications technology. Members of the focus group discussed the components of the items and were invited to collectively modify and validate a usable version of the instrument.

#### The COVID-19 Fear Scale

The Arabic-language adapted short version of the COVID-19 Fear Scale from Alyami et al was used [31]. This version has been translated and adapted into Arabic from the initial version of Ahorsu et al [32]. The scale assesses fear of COVID-19 using a one-dimensional factor tool divided into seven items, which are assessed on a 5-point Likert scale, ranging from 1 (strongly disagree) to 5 (strongly agree). Concomitant and confirmatory reliability and validity were examined on a set of Saudi participants.

The internal consistency of the Arabic version examined using Cronbach α was satisfactory (α=.88), with strong concurrent validity indicated by significant and positive correlations with the Hamilton Anxiety and Depression Scale (*r*=0.6). Likewise, examination of the factor structure according to Alyami et al [31] was adequate (comparative fit index [CFI]=0.995; root mean square error of approximation [RMSEA]=0.059; standardized root mean residual [SRMR]=0.024).

#### The 10-Item Perceived Stress Scale

An Arabic-language version of the 10-item Perceived Stress Scale (PSS-10) by Cohen et al [33], adapted by Almadi et al [34], was used to assess stress. The PSS-10 is divided into two subscales: the first assesses perceived psychological distress, while the second measures coping strategy. Scores are obtained on a 5-point Likert scale, ranging from 0 (never) to 4 (very often). The reliability and validity of the Arabic version of the PSS-10 presented a two-factor structure adequate for exploratory factor analysis, and their Cronbach α coefficients were .74 and .77, respectively. In addition, the test-retest reliability had an intracorrelation coefficient of 0.90.

For the purpose of our study, we considered only the related negative factor, which is distress; as such, the coping strategy was not taken into consideration.

#### The Arabic Internet Addiction Test

To measure internet addiction, we used the Arabic language–adapted scale from Hawi [35]. The Arabic version of the Internet Addiction Test (IAT) is an adapted version of the instrument originally developed by Young [36]. It consists of 20 items, each of which is scored on a 6-point Likert scale, ranging from 1 (strongly disagree) to 6 (strongly agree). The scale exhibits a unidimensional construct with robust psychometric properties: the goodness-of-fit indices demonstrated by the confirmatory factor analysis were all adequate (normed fit index [NFI]=0.96; CFI=0.98; Tucker-Lewis index [TLI]=0.98; goodness-of-fit index [GFI] and adjusted goodness-of-fit index [AGFI] above the recommended thresholds of 0.90). In particular, the internal consistency examined using the classical Cronbach α statistical index was satisfactory (α=.92).

### Statistical Tools

Data normality was tested by skewness and kurtosis tests during the exploratory phase, while multivariate normality was examined during the confirmatory phase. Asymmetry values greater than 7 or kurtosis values greater than 3 were judged to be non-Gaussian [37] and possessing low psychometric sensitivity [38]. In addition, the Mardia coefficient of multivariate normality was calculated during the confirmatory phase.

The exploratory analysis was carried out by unweighted least squares with a direct oblimin rotation. To assess whether the data were suitable for factor analysis, the sampling adequacy was examined by the Kaiser-Meyer-Olkin (KMO) statistic. According to the suggestions of Hair et al [39], the KMO value must be greater than 0.50 to accept the factorial solution. Furthermore, the chi-square value of the Bartlett sphericity test, which should be not significant, was calculated [40]. The factors were retained for eigenvalues greater than 1 and by examining the scree plot. In addition, an item was deleted if its factor loading was less than 0.5 [39-41]. The scale relationships have been examined through Pearson correlation tests between the SMDS-12, the COVID-19 Fear Scale, and the PSS-10.

First-order confirmatory factor analyses were performed to examine the factor structure of the instrument. The reliability of the instrument was examined by evaluating three internal consistency indices simultaneously: McDonald ω, Cronbach α, and Gutmann λ6. Convergent validity and discriminant validity were assessed, respectively, by calculating the average variance extracted (AVE) and comparing the square roots of the AVE values to the correlation coefficients. The relationships between instrument dimensions, internet addiction, and mental health parameters were assessed by the Pearson correlation matrix.

Descriptive statistical analyses of the factor structure were performed with SPSS for Windows, version 26 (IBM Corp), and Amos software for Windows, version 23 (IBM Corp). Internal consistency indices were calculated using JASP open source software, version 0.8.5 (JASP Team).

## Results

### Exploratory Factor Analysis

Table 2 shows the descriptive statistics, with means and standard deviations; the skewness and kurtosis coefficients of normality; and the lambda factor loadings. The coefficients of normality support the normality of the distributions.

The results indicate that the SMDS-12 was appropriate for proceeding with factor analysis (KMO=0.89; Bartlett test of sphericity=2988.98; *df*=66; P<.001). Exploratory factor analysis indicated a three-factor solution (eigenvalues were 5.45, 2.004, and 1.39 for the first, second, and third factor, respectively), explaining up to 73.72% of the total variance, with items having lambda factor loadings ranging from 0.73 to 0.85. The first factor explained 45.42% of the total variance, the second factor explained 16.70% of the variance, and the last factor explained 11.60% of the variance. In addition, the examination of the scree plot confirms the three-factor solution; a distinct change in the slope can be seen in the plot in Multimedia Appendix 1.

**Table 2:** Exploratory factor analysis of the 12-item Social Media Disinformation Scale (SMDS-12) (n=437).

SMDS-12 item No.	Mean (SD)	Skewness	Kurtosis	Lambda factor loading
1	2.94 (1.25)	0.02	2.94	1.25
2	2.95 (1.21)	0.04	2.95	1.21
3	2.89 (1.17)	0.00	2.89	1.17
4	2.83 (1.18)	0.11	2.83	1.18
5	2.76 (1.09)	0.10	2.76	1.09
6	2.80 (1.13)	0.12	2.80	1.13
7	2.65 (1.11)	0.15	2.65	1.11
8	2.64 (1.04)	0.07	2.64	1.04
9	2.45 (1.12)	0.31	2.45	1.12
10	2.45 (1.12)	0.23	2.45	1.12
11	2.42 (1.11)	0.27	2.42	1.11
12	2.41 (1.06)	0.31	2.41	1.06

### Confirmatory Factor Analysis

Before proceeding with the confirmatory factor analysis, univariate and multivariate tests of normality were performed. The results indicate that the item distribution followed a Gaussian distribution (Table 3), while the Mardia coefficient of multivariate normality indicated a value of 7.98 with a critical ratio of 4.55. These results suggest that multivariate normality was violated; on the other hand, the Mardia coefficient is sensitive to the size of the sample.

**Table 3:** Confirmatory factor analysis of the 12-item Social Media Disinformation Scale (SMDS-12) (n=437).

SMDS-12 item No.	Mean (SD)	Skewness	Critical ratio	Kurtosis	Critical ratio
1	3.16 (1.16)	–0.1	–0.5	–0.7	–3.1
2	3.20 (1.12)	–0.2	–1.4	–0.7	–2.9
3	3.12 (1.08)	–0.1	–0.7	–0.6	–2.5
4	3.05 (1.11)	0.0	–0.2	–0.7	–2.8
5	2.88 (1.13)	0.0	–0.3	–0.8	–3.4
6	2.91 (1.13)	0.1	0.6	–0.8	–3.2
7	2.80 (1.08)	0.1	0.7	–0.6	–2.7
8	2.78 (1.05)	0.1	0.7	–0.6	–2.7
9	2.43 (1.14)	0.27	2.85	–0.85	–4.57
10	2.43 (1.13)	0.28	3.02	–0.82	–4.39
11	2.35 (1.11)	0.33	3.58	–0.78	–4.21
12	2.38 (1.10)	0.29	3.11	–0.85	–4.58

**Figure 1:**
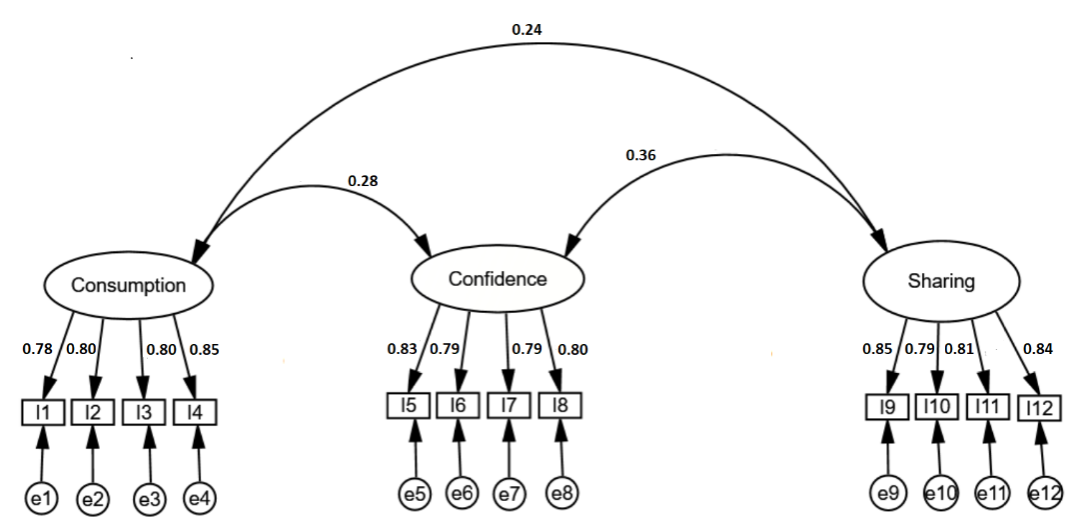
The final confirmatory factor analysis (CFA) of the 12-item Social Media Disinformation Scale. Factor correlation coefficients are 0.24 (between consumption and sharing), 0.28 (between consumption and confidence), and 0.36 (between confidence and sharing). Factor loadings range from 0.78 to 0.85. e1 to e12 represent the error variance for each item (I). CFA statistics: χ^2^_51_=62.5, *P*<.001; χ^2^/df=1.2; goodness-of-fit index=0.977; adjusted goodness-of-fit index=0.965; Tucker-Lewis index=0.995; comparative fit index=0.996; root mean square error of approximation=0.023 (90% CI 0-0.04); standardized root mean residual=0.036.

Figure 1 shows an overview of the model of the confirmatory factor analysis for the SMDS-12; following guidelines and recommendations [40,41], which suggest that a factorial weight greater than 0.71 can be considered to be excellent, we note that all items adequately contributed to the pre-established theoretical constructs. The confirmatory factor analysis results provided evidence for the three-factor structure of the SMDS-12. The factor loadings were acceptable and good (range 0.78 to 0.85).

The chi-square value obtained (χ^2^_51_=62.5; *P*<.001) may be due to the size of the sample. However, the chi-square divided by degrees of freedom value (χ^2^/df=1.2) respects the usual recommended threshold. The values of GFI and AGFI are 0.977 and 0.965, respectively. These two values must be greater than or equal to 0.90. In addition, the two indices TLI and CFI tend toward 1 and respect the threshold value of 0.95. Finally, the error indices—RMSEA=0.023 (90% CI 0-0.04) and SRMR=0.036—show that the measurement errors are tolerable.

### Reliability

The internal consistency and reliability of the three scale factors were calculated by the three indices: McDonald ω, Cronbach α, and Gutmann λ6. Examination of the indices for the three components of the scale yielded values greater than or equal to 0.80. This provides evidence for the internal consistency of the scale. Likewise, a good internal consistency was supported by the Cronbach α indices, which had values of .89, .88, and .88 for the consumption, confidence, and sharing subscales, respectively, as well as by the Gutmann λ6 coefficients, which were greater than or equal to 0.84. In addition, the corrected item-total correlation was calculated for each latent variable. The results show that the values were adequate, since they were located between 0.72 and 0.78 for the first component (ie, consumption), between 0.70 and 0.76 for the second component (ie, confidence), and between 0.73 and 0.76 for the last component (ie, sharing). These results confirm that the instrument has good reliability (Table 4). The internal consistency of the component is considered good if the value is equal to or greater than 0.70 [41].

**Table 4:** Internal consistency of the 12-item Social Media Disinformation Scale (SMDS-12).

Latent variable and SMDS-12 item No.		Corrected item-total correlation	McDonald ω		Cronbach α		Gutmann λ6	
**Consumption**				0.89		.89		0.86
	1	0.75						
	2	0.72						
	3	0.76						
	4	0.78						
**Confidence**				0.88		.88		0.85
	5	0.76						
	6	0.73						
	7	0.70						
	8	0.74						
**Sharing**				0.88		.88		0.85
	9	0.76						
	10	0.75						
	11	0.73						
	12	0.74						

### Construct Validity

#### Convergent Validity

The convergent validity was assessed following the Fornell-Larcker criterion [42] by the calculation of the AVE. AVE values above 0.7 are considered very satisfactory, whereas a level of 0.5 is considered acceptable. The AVE values were 0.67 for consumption, 0.64 for confidence, and 0.67 for sharing.

#### Discriminant Validity

Discriminant validity is ensured when the variance shared by two different latent variables is less than the variance shared by the latent variable and its indicators (ie, items). This implies that the square root of the AVE must be greater than all correlations between latent variables. The comparison of the square roots of the AVE values presented on the diagonal of the matrix (Multimedia Appendix 2) with the correlation coefficients shows that the discriminant validity of the scale was adequate.

The square roots of the AVE values for consumption, confidence, and sharing were 0.82, 0.80, and 0.81, respectively. The comparison of each AVE value with correlation coefficients with the other constructs shows that they were of higher value.

### Relationship Between the Credibility of Disinformation and Mental Health During the COVID-19 Pandemic

The correlation matrix (Table 5) provided positive, significant, and moderate associations between the dimension of consumption and internet addiction (*r*=0.22), perceived stress (*r*=0.16), and the fear of COVID-19 (*r*=0.21). For the confidence subscale, a moderate correlation was demonstrated with internet addiction (*r*=0.34), while the correlations with perceived stress and fear of COVID-19 were 0.14 and 0.23, respectively. The sharing dimension resulted in a correlation coefficient 0.19 with internet addiction and lower coefficient values for perceived stress (*r*=0.093) and fear of COVID-19 (*r*=0.16).

**Table 5:** Correlation matrix between the 12-item Social Media Disinformation Scale subscales and mental health parameters related to COVID-19.

Variable		Consumption	Confidence	Sharing	Internet addiction	Perceived stress	Fear of COVID-19
**Consumption**							
	*r*	1	0.35^a^	0.27^a^	0.22^a^	0.16^a^	0.21^a^
	P value	—^b^	<.001	<.001	<.001	<.001	<.001
**Confidence**							
	*r*	0.35^a^	1	0.33^a^	0.34^a^	0.14^a^	0.23^a^
	P value	<.001	—	<.001	<.001	<.001	<.001
**Sharing**							
	*r*	0.27^a^	0.33^a^	1	0.19^a^	0.093^c^	0.16^a^
	P value	<.001	<.001	—	<.001	.014	<.001
**Internet addiction**							
	*r*	0.22^a^	0.34^a^	0.19^a^	1	0.14^a^	0.21^a^
	P value	<.001	<.001	<.001	—	<.001	<.001
**Perceived stress**							
	*r*	0.16^a^	0.14^a^	0.093^c^	0.14^a^	1	0.33^a^
	P value	<.001	<.001	.014	<.001	—	<.001
**Fear of COVID-19**							
	*r*	0.21^a^	0.23^a^	0.16^a^	0.21^a^	0.33^a^	1
	P value	<.001	<.001	<.001	<.001	<.001	—

^a^The correlation is significant at a significance level of .01 (two-tailed).

^b^Not applicable.

^c^The correlation is significant at a significance level of .05 (two-tailed).

## Discussion

### Principal Findings

The objective of this study was to develop and test the psychometric properties of the SMDS-12 measurement scale to assess consumption, confidence, and sharing of information related to COVID-19 by social media users. The 12-item scale was initially tested through exploratory factor analysis.

The test supported the three-factor structure; in addition, no items were removed from the measurement scale. Subsequently, confirmatory factor analysis confirmed the robustness of the measurement tool. The results also supported the construct validity of the scale by its convergent and discriminant validity, both of which were adequate. The reliability of the instrument examined by means of three internal consistency indices and the corrected item-total correlation demonstrated that the three dimensions of the instrument are reliable.

The correlation between the three dimensions of the instrument with the internet addiction scale and mental health factors showed positive associations, which lay in a range from small, for the relationship of the sharing dimension with stress, to moderate, for the association of the other two factors with internet addiction, perceived stress, and fear of COVID-19.

Regarding the links between the consumption of disinformation and internet addiction, similar results have been reported by Priego-Parra et al [43]. The authors found that internet addiction and overexposure to rapidly spreading disinformation are associated with anxiety and depression. In addition, internet addiction resulting in obtaining information about COVID-19 has increased stress and anxiety levels.

Furthermore, in other studies of COVID-19 related to disinformation spread on social media [44-48], aimed at identifying the prevalence and factors associated with the concept, disinformation was shown to be linked to demographic variables, such as age, gender, and academic level. Moreover, consistent with our findings, misinformation beliefs were significantly associated with fear of COVID-19 in addition to other variables, such as lower levels of health education, trust in government, and confidence in science.

During the COVID-19 pandemic, internet addiction and the use of social media in particular have increased significantly [44-48]. Also, time spent on the internet was associated with sharing misinformation related to the context of the illness [44-49].

Moreover, some studies [50-52] examined the association between social media and mental health linked to the COVID-19 pandemic. The results showed that social media use was linked to depression, and excessive social media use led to mental health issues.

Our findings are also in line with a pilot study by Zhong et al [20], which examined the possible association between social media use and the mental health toll linked to the COVID-19 pandemic in China. This study found that social media use was linked to both depression and secondary trauma, which also predicted a change in health behavior.

On the contrary, in a cross-sectional survey by Agley and Xiao [14], COVID-19–related information sharing behaviors were clustered, and four belief profiles emerged from the analysis. A total of 70% of the subjects surveyed believed in scientifically accepted theories (ie, zoonotic origin of the outbreak) and not in conspiratorial theories. Other profiles disagreed with the zoonotic explanation, and instead believed in misinformation, although to varying degrees. Briefly, trust in science was a strong and significant predictor of news sharing behavior.

Regarding the acquisition of disinformation and the subsequent sharing of this information, Chua and Banerjee [53] showed that gullible users had a greater propensity to share health rumors online. For that reason, Li and Sakamoto [54] suggested that exposing individuals to collective opinion measures may reduce the tendency to share false health messages. To explain the mechanism, the theory of cultural attraction can be utilized. Indeed, this theory postulates that the spread of rumors results from psychological pull factors. The reasons for the propagation of this false information are mainly due to the recruitment of cognitive pull factors that are likely to increase social interactions [55]. Indeed, on these platforms, content creators produce their works with a strong psychological appeal to encourage users to react to them and increase their audiences.

This highlights the need for much more research into the cultural, psychological, and social characteristics of users who trust and disseminate this content on social media. In particular, it is crucial to better understand the roles of thinking and belief systems. For example, they should also be explored in empirical studies, in particular, relying on mathematical models based on big data and artificial intelligence. This would be of paramount importance, given the potential impact of COVID-19–related misinformation on the public health measures implemented to curb the pandemic [56-61].

### Conclusions and Recommendations

The results of this study provided a first demonstration for assessing behaviors related to use, consumption, and sharing of information related to COVID-19 on social media. The SMDS-12 exhibited acceptable psychometric properties and can be utilized in Tunisia and other Arabic countries to explore user engagement with social media, credibility of information, and interaction with information in terms of sharing. Furthermore, the instrument could be translated, culturally validated, and utilized by other scholars from other countries.

### Limitations of the Study

The main limitation of this study is the lack of concurrent validity testing of the instrument with similar instruments. In addition, the instrument has only been tested on a single population living in a single country. Also, the study was observational and not interventional; it did not investigate ways that could reduce credibility and counteract the sharing of rumors and misinformation. Another limitation relates to the study population, as the data were collected from a group of Tunisian social media users. Although we have confirmed the validity and reliability of the measurement instrument for these participants, a certain specificity linked to the cultural context does not allow for the generalization of the results.

## Appendix

Multimedia Appendix 1 Scree plot for the principal component analysis of the 12-item Social Media Disinformation Scale (SMDS-12).

Multimedia Appendix 2 Discriminant validity of the 12-item Social Media Disinformation Scale (SMDS-12) subscales.

## Abbreviations

AGFI: adjusted goodness-of-fit index
AVE: average variance extracted
CFI: comparative fit index
GFI: goodness-of-fit index
IAT: Internet Addiction Test
IDRC: International Development Research Centre
KMO: Kaiser-Meyer-Olkin
NFI: normed fit index
NPI: nonpharmaceutical intervention
PSS-10: 10-item Perceived Stress Scale
RMSEA: root mean square error of approximation
SMDS-12: 12-item Social Media Disinformation Scale
SRMR: standardized root mean residual
TLI: Tucker-Lewis index
UNESCO: United Nations Educational, Scientific and Cultural Organization
