# Supplementary figures and images for: Development of the 12-Item Social Media Disinformation Scale and its Association With Social Media Addiction and Mental Health Related to COVID-19 in Tunisia: Survey-Based Pilot Case Study
Source: JMIR Form Res. 2021 Jun 9;5(6):e27280. doi: 10.2196/27280 (PMC8191730; doi:10.2196/27280)

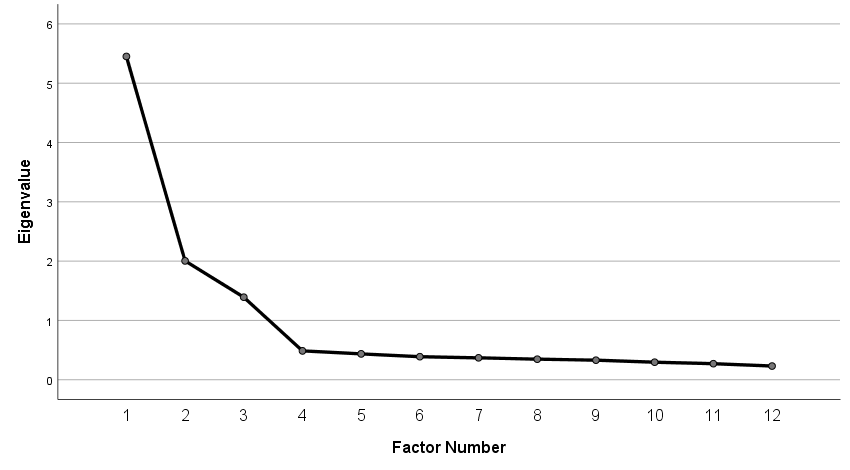

Supplement: Multimedia Appendix 1 [file formative_v5i6e27280_app1.png]
