# Supplementary material for: Development of the 12-Item Social Media Disinformation Scale and its Association With Social Media Addiction and Mental Health Related to COVID-19 in Tunisia: Survey-Based Pilot Case Study
Source: JMIR Form Res. 2021 Jun 9;5(6):e27280. doi: 10.2196/27280 (PMC8191730; doi:10.2196/27280)
Supplement: Multimedia Appendix 2 [file formative_v5i6e27280_app2.docx]

**Multimedia Appendix 2. Discriminant validity of the 12-item Social Media Disinformation Scale (SMDS-12) subscales.**

| Latent variables | Consumption | Confidence | Sharing |
| --- | --- | --- | --- |
| Consumption | **0.82** | 0.33 | 0.30 |
| Confidence | 0.33 | **0.80** | 0.38 |
| Sharing | 0.30 | 0.38 | **0.81** |
